# Supplementary material for: Simple Muscle Architecture Analysis (SMA): An ImageJ macro tool to automate measurements in B-mode ultrasound scans
Source: PLoS One. 2020 Feb 12;15(2):e0229034. doi: 10.1371/journal.pone.0229034 (PMC7015391; doi:10.1371/journal.pone.0229034)
Supplement: S1 Supporting Information — (PDF) [file pone.0229034.s001.pdf]

Supporting information

Installation instructions for SMA (Simple Muscle Architecture Analysis): An ImageJ/Fiji based macro for automated analysis of B-mode ultrasound images.

Step 1: Download the Fiji software using the following link. **Please read the warning on this page about where to install Fiji, as this can affect your ability to get updates**:
https://imagej.net/Fiji/Downloads

Step 2: After downloading and running Fiji, you need to gather a few dependencies. First, click *Help* from the dropdown menu in Fiji, then choose *Update.* In the Updater window, select *Manage update sites*. In the window that appears, you will notice that some boxes are already ticked. This means that if/when an update is released for the corresponding programme, your machine will automatically install it at the first opportunity.

Scroll down the list and tick the *BIG-EPFL* and *Biomedgroup* update sites. Then, click the *Add update site* button and scroll to the bottom of the list, where a new update site has been added. Modify the details of the new site as follows (to modify a section, double-click it):
Name: SMA
URL: http://sites.imagej.net/SMA
Host: webdav:SMA

After entering the details, make sure that the box to the left of the SMA text is ticked. Click *Close* once you have finished editing. Click *Apply changes* to confirm the installation. Close Fiji and re-open it. You are now ready to use SMA.

Optional step 3 (recommended): It is convenient to add the SMA macro to the list of available plugins so that you can run SMA from the dropdown menu. Otherwise you'll need to open the SMA_1_6.ijm file from the *Fiji macros* folder, and run it manually.

On Mac, SMA would automatically be added to the *Plugins* dropdown list. To add SMA to the *Plugins* dropdown list on Windows, select *Plugins* from the dropdown menu, then *Install*, and choose the SMA_1_6.ijm file (the X will depend on what version you have), which you can find by double-clicking your main *Fiji* folder, then double-clicking the *macros* folder, which should contain the target file. Select it and choose *open*. Finally, you will see a different window asking where you would like to save this file. Choose the Fiji plugins folder (should be the default) and click *save*. The SMA macro should now appear at the bottom of the dropdown *Plugins* list.

Optional step 4: To analyse movie frames (see limitations in the companion article) with SMA, movies must first be imported into Fiji and converted into image sequences (NB: movies can also be converted in advance using some other software). This is possible if the update site for the *FFMEG* plugins is added (follow the procedure described above).

A movie file can then be imported by clicking *File > Import > Movie (FFMPEG)*, selecting the movie and accepting the default *Import options*. Once imported, the movie can be converted into an image sequence in several ways.
One way is to click *Image > Stacks > Tools > Make Substack…* and then enter the range of frames to be exported. The obtained image stack can then be archived by clicking *File > Save as >Image Sequence,* and selecting the appropriate file format and saving location.
